# Non-Vitamin K Oral Anticoagulants in Adults with Congenital Heart Disease: A Systematic Review

**DOI:** 10.3390/jcm9061794

**Published:** 2020-06-09

**Authors:** Nikolaos Stalikas, Ioannis Doundoulakis, Efstratios Karagiannidis, Emmanouil Bouras, Anastasios Kartas, Alexandra Frogoudaki, Haralambos Karvounis, Konstantinos Dimopoulos, George Giannakoulas

**Affiliations:** 1 First Department of Cardiology, AHEPA Hospital, Aristotle University of Thessaloniki, 54 636 Thessaloniki, Greece; nstalik@gmail.com (N.S.); doudougiannis@gmail.com (I.D.); stratoskarag@gmail.com (E.K.); tkartas@gmail.com (A.K.); hkarvounis@gmail.com (H.K.); 2 Department of Internal Medicine, General Hospital of Edessa, 582 00 Proastio, Greece; 3 Department of Cardiology, 424 General Military Training Hospital, 54 124 Thessaloniki, Greece; 4 Department of Hygiene, Social-Preventive Medicine & Medical Statistics, Medical School, Aristotle University of Thessaloniki, 54 124 Thessaloniki, Greece; mpman33@gmail.com; 5 Second Department of Cardiology, Attikon University Hospital, University of Athens, 124 62 Athens, Greece; afrogou@otenet.gr; 6 Adult Congenital Heart Centre and National Centre for Pulmonary Hypertension, Royal Brompton Hospital and Imperial College, London SW3 6NP, UK; k.dimopoulos@rbht.nhs.uk

**Keywords:** congenital heart defects, anticoagulants, thromboembolism, hemorrhage, treatment efficacy, patient safety

## Abstract

Adults with congenital heart disease (ACHD) experience more thromboembolic complications than the general population. We systematically searched and critically appraised all studies on the safety and efficacy of non-vitamin-K oral anticoagulants (NOACs) in adult patients with various forms of congenital heart disease. PubMed and the Cochrane Central Register of Controlled Trials (CENTRAL) were used, with duplicate extraction of data and risk of bias assessment. The Newcastle-Ottawa quality assessment scale was used to assess study quality. Three studies fulfilled the inclusion criteria and were analyzed. The total number of participants was 766, with a total follow-up of 923 patient-years. The majority of patients (77%) received a NOAC for atrial arrhythmias, while the remainder were prescribed NOACs for secondary (19%) or primary (4%) thromboprophylaxis. The annual rate of thromboembolic and major bleeding events was low: 0.98% (95% CI: 0.51–1.86) and 1.74% (95% CI: 0.86–3.49) respectively. In Fontan patients, the annual rate of thromboembolic and major bleeding events was 3.13% (95% CI: 1.18–8.03) and 3.17% (95% CI: 0.15–41.39) respectively. NOACs appear safe and effective in ACHD without mechanical prostheses. Additional studies are, however, needed to confirm their efficacy in complex ACHD, especially those with a Fontan-type circulation.

## 1. Introduction

Adults with congenital heart disease (ACHD) are a rapidly growing population, both in number and age, and are at increased risk of thromboembolic complications, especially strokes, compared to the general population [1,2,3,4]. Thromboembolic events are multifactorial in this population and are influenced by the complexity of the congenital heart disease (CHD), presence of cyanosis, repair status, prosthetic valves and other material, and residual lesions [4,5,6,7,8,9]. Moreover, supraventricular arrhythmias are common in ACHD patients, and the rise in prevalence is strongly related to aging, as well as to the number and type of previous interventions, and the effects of long-standing hemodynamic lesions [10,11].

With limited evidence to support clinical decision-making in ACHD, current indications for the use of anticoagulants in these patients are mainly based on expert opinion. The Pediatric and Congenital Electrophysiology Society and Heart Rhythm Society (PACES/HRS) Expert Consensus Statement on the Recognition and Management of Arrhythmias in Adults with Congenital Heart Disease advises long-term anticoagulation in patients with recurrent or sustained intra-atrial reentrant tachycardia, or atrial fibrillation (AF) in the presence of moderate or complex CHD (class IIa and I indication respectively) [12]. The anticoagulants of choice in these situations are vitamin K antagonists (VKA), as data on the safety and efficacy of non-vitamin K oral anticoagulants (NOACs) are limited. This consensus statement did, however, recommend the use of NOACs in patients with intra-atrial reentrant tachycardia or atrial fibrillation and simple nonvalvular CHD (class IIb indication). The routine administration of NOACs in patients with a Fontan circulation was felt to be contraindicated (class III indication), as evidence is lacking. Moreover, the CHA_2_DS_2_-VASc (congestive heart failure, hypertension, age 65–74 years, diabetes mellitus, sex-female, (1 point for the presence of each) and age >75 years, Stroke/TIA (2 points); range from 0 to 9) and HAS-BLED (uncontrolled systolic blood pressure >160 mm Hg, abnormal renal and/or liver function, previous stroke, bleeding history or predisposition, labile international normalized ratios, elderly, and concomitant drugs and/or alcohol excess (1 point for the presence of each); range from 0 to 9) scores, which are validated to assess stroke and bleeding risk respectively in patients with nonvalvular AF in the general population, are of unclear value in the ACHD population [13,14,15].

Recently, new evidence has emerged on the role of NOACs in ACHD patients with an indication for thromboprophylaxis [16,17]. Indeed, the potential advantages of NOACs in this young population are multiple, including ease-of-use and avoidance of periods of over- or under-anticoagulation in patients that are likely to remain anticoagulated for very long periods. We systematically collected all available evidence on the use of NOACs in various types of ACHD, aiming to assess the safety and efficacy of this medication in this population.

## 2. Materials and Methods

This systematic review followed the recommendations of the PRISMA statement [18]. All research was conducted according to a protocol registered in the PROSPERO database (PROSPERO registration number: CRD42020158227 review protocol available in https://www.crd.york.ac.uk/prospero/display_record.php?RecordID=158227).

### 2.1. Search Strategy

PubMed and Cochrane Central Register of Controlled Trials (CENTRAL) were used for all searches from inception to November 2019. We also searched for randomized clinical trials in the U.S. National Library of Medicine’s registry of clinical trials [19]. A basic search strategy was developed for PubMed and modified accordingly for other research engines. We also searched Prospero for a similar systematic review in progress, to avoid duplication. The PubMed and Cochrane Central strategy is presented in the Appendix A. The search was conducted by two independent investigators (NS, ID).

### 2.2. Eligibility Criteria

We included studies that assessed the efficacy and/or safety of NOACs in ACHD patients with an indication of thromboprophylaxis (atrial arrhythmias, primary, or secondary thromboprophylaxis). In this systematic review, ACHD patients were classified according to the severity of their congenital heart defect, as stated in the Task Force 1 of the 23rd Bethesda Conference [20]. Safety was reported as the rate of major bleeding events, in accordance with the criteria provided by the International Society on Thrombosis and Hemostasis [21]. Animal studies, case reports, reviews, and studies in children were excluded (Table 1).

### 2.3. Selection of Studies

All studies were imported into a reference management software. After removal of duplicates, the two investigators (NS, ID) independently screened all titles. Studies that were eligible based on the title and abstract, were screened as full text by the reviewers. There was no disagreement between the two reviewers regarding abstract selection, so a third reviewer (EK) was not necessary.

### 2.4. Quality Assessment

The quality of the eligible studies was evaluated by two independent reviewers (NS, ID) using a version of the Newcastle-Ottawa scale (NOS, Universities of Newcastle, Australia and Ottawa, Canada) quality assessment scale for observational studies, as only cohort studies were included in this systematic review [22]. The risk of bias assessment was carried out by the same reviewers (NS, ID), with no disagreements between them.

### 2.5. Data Extraction

The two reviewers independently extracted the following data: number of patients in the study, study population, mean age, gender, type of congenital heart disease, type of surgery (in case of operated CHD), indication for anticoagulation, type of oral anticoagulation administered, mean duration of follow up in patient-years, incidence of thromboembolic events, severe bleeding events, and CHA_2_DS-VA_2_Sc and HAS-BLED scores.

### 2.6. Data Synthesis–Statistics

Although the studies had inherent differences (such as different case mix and different follow-up periods), the population (ACHD) and the core of the intervention (i.e., use of NOACs in ACHD patients with an indication of thromboprophylaxis) was similar. Hence, we presented pooled statistics. We performed a random-effects meta-analysis using a generalized linear mixed model [23] and Clopper–Pearson confidence intervals for individual studies [24]. We preferred a generalized linear mixed model over a Freeman–Tukey double arcsine transformation due to potential methodological concerns regarding the back-transformation of meta-analysis results with the latter when individual study sizes differ significantly [25]. The analysis was performed in R version 3.6.0 [26], utilizing the meta package [27].

## 3. Results

**Figure 1:**
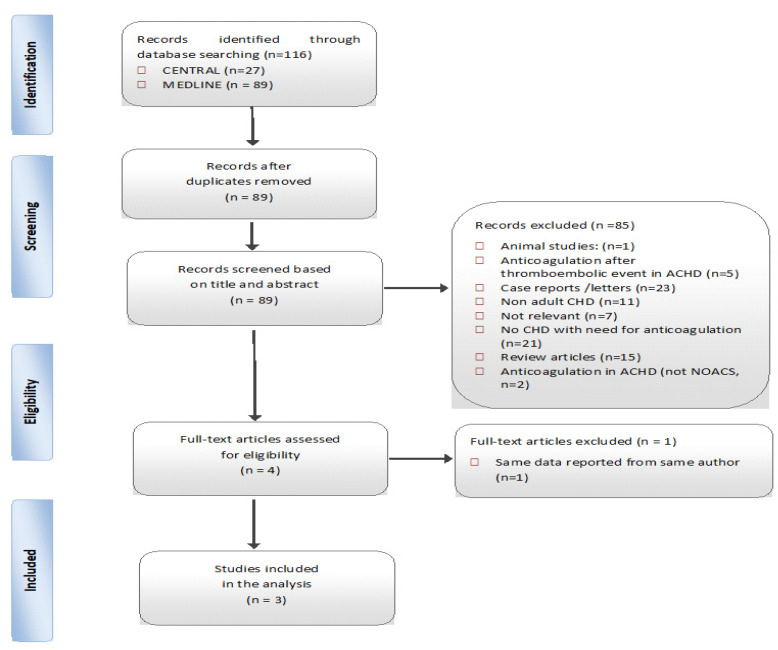
Summary of evidence of search and selection. Abbreviations: ACHD: adults with congenital heart disease; CHD: congenital heart disease, NOACs: non-vitamin-K oral anticoagulants.

A total of 89 studies were identified (69 on PubMed and 20 on CENTRAL). Of these, 85 were excluded based on their title and abstract (Figure 1, PRISMA flow diagram of the methodology). Of the four studies that were assessed for eligibility by full-text screening, one was excluded. Ultimately, three studies fulfilled the inclusion criteria and were assessed for quality. All studies were written in English. Details of the quality assessment process and results are presented in Table 2.

### 3.1. Cumulative Data and Individual Study Characteristics

The characteristics of the included studies and their endpoints are depicted in Table 3. The total number of participants in the three included studies was 766, and the total duration of follow-up was 923.1 patient-years. ACHD patients of various types and complexity were included, but the primary endpoint was similar. The vast majority (77%) of patients received a NOAC due to atrial arrhythmias (Table 4), followed by 19% of patients who were prescribed a NOAC for secondary thromboprophylaxis (following pulmonary embolism, deep vein thrombosis, systemic embolism, intracardiac thrombus, ischemic stroke, myocardial infarction due to paradoxical embolism). Only 4% of patients were prescribed NOACs for primary thromboprophylaxis (e.g., Fontan palliation or a cyanotic defect).

Pujol et al. [29] examined the use of NOACs in 215 ACHD patients. In this cohort, 44% of the total population had complex CHD, including 12 patients (5.6%) with a Fontan circulation, and 17 patients (7.9%) with chronic cyanosis. Most patients were anticoagulated with rivaroxaban (54.2%), followed by apixaban (32.2%), dabigatran (9.3%), and edoxaban (3.7%). Almost 1 out of 10 patients (*n* = 24), received concomitant treatment with aspirin, one was treated with clopidogrel, whereas three patients received dual antiplatelet treatment with aspirin and clopidogrel. The mean age was 48.4 ± 15.4 years and the mean follow up was 15.8 ± 15.8 months. Follow-up information was obtained using a detailed medical questionnaire that was mailed to all patients. Many patients received anticoagulation treatment for more than one indication (e.g., atrial arrhythmias and stroke/transient ischemic attack). In two-thirds of the patients (*n* = 143), the primary indication for anticoagulation was atrial arrhythmias (AF 69%, atrial flutter 31%), 54% received a NOAC for secondary thromboprophylaxis and 5.6% for primary thromboprophylaxis in the presence of a Fontan circulation. The CHA_2_DS_2_-VASc and HAS-BLED scores were calculated in all patients: 49.3% had a CHA_2_DS_2_-VASc ≥ 2, and 87.5% a HAS–BLED score ≤ 2. During follow-up, two thromboembolic events were reported in patients to whom a NOAC was prescribed for primary thromboprophylaxis and nine major bleeding events occurred (Table 5). The rate of thromboembolic and major bleeding events per patient-year was 0.7% and 3.1% respectively. For the few patients in this cohort who received concomitant antiplatelet treatment, bleeding risk was not found to be increased in univariate analysis (HR 3.18, CI (0.89–11.4), *p* = 0.08). Finally, multivariate analysis showed that renal disease, which was present in almost 4% of the total population, was identified as the only major risk factor for major bleeding (HR: 13.75, CI: 2.60–72.54, *p* = 0.002).

Georgekutty et al. [30] reported the first retrospective data on the efficacy and safety of NOACs in patients with a Fontan-type circulation (76% had a total cavopulmonary connection). In this study, 21 adult patients with Fontan circulation were prescribed a NOAC either for primary or secondary thromboprophylaxis (apixaban 63%, dabigatran 18.5%, rivaroxaban 18.5%). Specifically, 57% of patients received a NOAC due to atrial arrhythmias, 9.7% due to persistent right-to-left shunts, and 33.3% because of prior thrombotic events. All patients were treated with warfarin before NOAC initiation; the main reasons for the change in anticoagulant were either patient/provider preference, suboptimal international normalized ratio (INR) control, initiation of therapy elsewhere, or history of poor clinical follow-up. The CHA_2_DS_2_-VASc and HAS-BLED scores were calculated in all patients: a CHA_2_DS_2_-VASc ≥ 2 was present in 42.9% of patients, while all patients had a HAS-BLED score ≤ 2. During a cumulative follow-up of 316 patient-months, one thrombotic event occurred in a 21-year-old “failing Fontan” patient with protein-losing enteropathy, who developed deep vein thrombosis after 12.6 months of treatment with dabigatran. No major bleeding events were reported, but 10 patients experienced minor bleeding (minor bleeding event rate of 38% per patient-year). Finally, one death occurred in a patient with multiorgan system failure after a out-of-hospital cardiac arrest.

Yang et al. [28] reported on the results of the NOTE registry, the first international multicenter cohort study assessing the safety and efficacy of NOACs (rivaroxaban 43%; apixaban 39%; dabigatran 12%; edoxaban 7%) in ACHD patients. A quarter of patients (28%, *n* = 150) were previously on Vitamin K Antagonist (VKA) therapy and served as historic controls. The total duration of follow-up was 613 patient-years, with a median treatment duration of 1.0 (IQR 0.0–2.0) year, while in the VKA historical cohort this was 3.8 (IQR: 1.1–8.6) years. The investigators also assessed adherence to treatment and the effect of NOACs on quality of life (QoL). A total of 530 patients with ACHD of various complexities were included. Almost 15% of patients (*n* = 74) had a Fontan-type circulation, 65% of whom had a total cavopulmonary connection. A CHA_2_DS_2_-VASc ≥ 2 was present in 46% and an HAS-BLED ≤ 2 in 95%. Indications for anticoagulation included atrial arrhythmias (91%, *n* = 481, (AF 58%, atrial flutter 40% and atrial tachycardia 2%)), primary (3%, *n* = 17), and secondary thromboprophylaxis (6%, *n* = 32). All predefined endpoints were assessed by an expert investigator or site coordinator. There were six (1.1%) thromboembolic events reported, with a rate of 1.0% per year (95% CI: 0.4–2.0). All thromboembolic events occurred in patients with moderate or severe forms of ACHD, and 50% (*n* = 3) were in patients with a Fontan circulation. Major bleeding events occurred in seven patients, i.e., 1% per year (95% CI: 0.5–2.2) and 43% of major bleeds (*n* = 3) occurred in Fontan patients. Minor bleeding events were reported in 37 patients, with an estimated rate of 6.3% per year (95% CI: 4.5–8.5). In terms of adherence to treatment during follow-up, 80–93% of patients had sufficient adherence (assessed with the Morisky-8-questionnaire or using pharmacy interrogation data). Moreover, 33 prior VKA users reported an improvement in QoL in six out of eight domains (*p* < 0.05), while VKA-naïve patients (*n* = 39) reported no decline in QoL.

### 3.2. Cumulative Results

**Figure A1:**
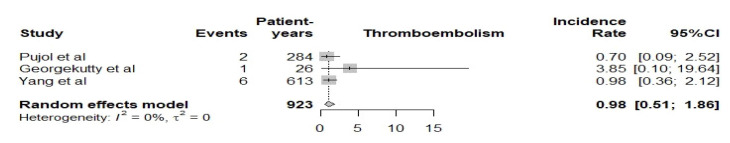
Forest plot of the incidence of thromboembolic events in ACHD patients.

**Figure A2:**
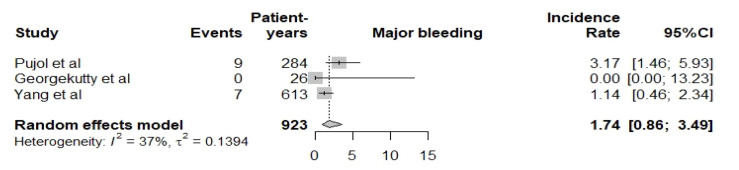
Forest plot of the incidence of major bleeding events in ACHD patients.

The annual incidence of thromboembolic and major bleeding events in the overall population was low: 0.98% (95% CI: 0.51–1.86, *n* = 9 thromboembolic events) and 1.74% (95% CI: 0.86–3.49, *n* = 16 major bleeding events) respectively (see Appendix A, Figure A1 and Figure A2). The majority of thromboembolic events (4/9) and major bleeds (3/16) occurred in patients with a Fontan circulation (*n* = 107, Table 5 and Table 6), corresponding to an annual rate of thromboembolic events of 3.13% (95% CI: 1.18–8.03) and an annual rate of major bleeding events of 3.17% (95% CI: 0.15–41.39). Of the four thromboembolic events in Fontan patients, only one was systemic (ischemic stroke); the remainder were pulmonary embolisms (*n* = 1), deep vein thrombosis (*n* = 1), and intracardiac thrombosis (*n* = 1). The most commonly used NOAC was rivaroxaban (45%), followed by apixaban (38%), dabigatran (12%) and edoxaban (5%). Of 9 thromboembolic events, 2 occurred in patients treated with rivaroxaban (2/349), 3 to those on dabigatran (3/89), and 4 to those on apixaban (4/293). Most major bleeding events (9 out of 16) occurred in patients treated with rivaroxaban (9/349), followed by those on apixaban (*n* = 6 major bleeds, 6/293), one major bleed occurred in a patient anticoagulated with edoxaban (1/35) and none in those treated with dabigatran (0/89). Since the event rate was low, direct comparisons between the different effects of NOACs or anatomical subgroups were not performed. No fatal event reported related to thromboembolism or major bleeding during follow-up. Of note, the median CHA_2_DS_2_-VASc score of patients who suffered a thromboembolic event was 1.0.

## 4. Discussion

This is the first systematic review of studies that has investigated the efficacy and safety of NOACs in ACHD patients. The few studies available appear reassuring with regards to the safety of NOACS in the ACHD population, with a low major bleeding rate in patients with both simple and complex lesions. The evidence regarding the efficacy of NOACS in this population also appears encouraging, with a low thromboembolic event rate overall. The efficacy and safety data of NOACs in ACHD collected in this study do not appear to differ significantly from what is reported for VKAs in this population. Indeed, two retrospective studies in ACHD patients with atrial arrhythmias treated with VKAs reported a 1.1–1.3% annual rate of thromboembolic events and 0.8–4.4% annual rate of major bleeding events [14,31]. However, more data are needed for Fontan patients, who appear to have a higher event rate than the remainder. 

The majority of patients included in the three analyzed studies had CHD of significant complexity, with one study (Georgekutty et al.) including only adults with a Fontan circulation. In the largest study (Yang et al.), 85.1% of patients had CHD of moderate or high complexity, reflecting the population of patients with indications for anticoagulation in real life. Despite the high prevalence of complex CHD in the population included in this metanalysis, the incidence of thromboembolic events whilst on NOACs was low, with the exception perhaps of Fontan patients. Indeed, almost half of thromboembolic events during follow-up occurred in patients with a Fontan circulation. This can manifest as thrombosis of the Fontan and pulmonary circulation, with its slow, non-pulsatile flow, endothelial damage, hypercoagulability, or paradoxical emboli to the systemic circulation through a fenestration or collateral circulation [32,33,34,35]. NOACs may be insufficient to prevent thrombosis in some Fontan patients, especially those with older types of Fontan operations (atriopulmonary Fontan), in whom a severely enlarged right atrium, a low cardiac output state, and frequent arrhythmias may require a stronger anticoagulation regime. However, in a recent meta-analysis of Fontan patients receiving VKA (i.e., the current standard-of-care), the overall incidence of thromboembolism reached 10% [36].

ACHD patients requiring anticoagulation are often young, and any decision on the type of anticoagulant prescribed should take into account their thrombotic risk, but also their life expectancy and the impact it could take on their lifestyle. NOACs possess many advantages in contrast to VKAs. They have a rapid onset of action, fewer drug and food interactions, predictable pharmacokinetics, and a shorter half-life [37]. Most importantly, NOACs do not require laboratory monitoring, making them more acceptable for younger active patients [38]. A recent cross-sectional study demonstrated high non-adherence rates or discontinuation of warfarin (26% and 17% respectively) in 127 young patients (mean age 31.2 years) after valve replacement surgery for rheumatic heart disease with a mean time since surgery of 3.7 years [39]. In general, adherence to NOACs is superior to VKAs [40], with one out of three patients who start warfarin for atrial fibrillation discontinuing treatment after one year of therapy [41,42,43,44]. Adherence to NOACs was also good (≥ 80%) during the two years of follow-up in the NOTE registry. Optimal long-term adherence to treatment is likely to translate into a lower thromboembolic event rate, especially in younger ACHD patients who may need anticoagulation for decades.

Sample size restrictions and differences in population characteristics aside, the mean annual thromboembolism (0.98%) and annual major-bleeding rates (3.13%) reported in our study did not exceed those of any of the NOAC landmark trials [45,46,47]. Participants in our analyzed studies had low median HAS-BLED and CHA_2_DS_2_-VASc scores, which may explain the low overall incidence of thromboembolic and bleeding events. However, the mechanisms behind thrombotic events in ACHD patients often differ to those of the general population and patients with acquired heart disease, and there is no firm evidence on the accuracy of these scores in the ACHD population. In a retrospective cohort study of 482 ACHD patients with atrial arrhythmias treated with oral anticoagulation or antiplatelet therapy, the CHA_2_DS_2_-VASc was unable to accurately predict thromboembolic events [14], while the severity of the underlying CHD (50% with severe CHD) was associated with the thromboembolic risk. In another retrospective cohort study of 158 patients with atrial arrhythmias who were not anticoagulated, thromboembolic events were also observed in patients with low or intermediate CHA_2_DS_2_-VASc scores [15]. The CONCOR registry, which assessed the rate of thromboembolic and bleeding events in 229 ACHD patients with atrial arrhythmias, reported that ACHD patients with a HAS-BLED score ≥2 receiving VKAs had an almost three-fold increased risk of a bleeding events compared to patients with a score <2 [31]. This suggests that the HAS-BLED score may have a role in the ACHD population, although more data are needed to confirm this finding.

This systematic review has several limitations. Only three observational studies met our inclusion criteria on the absence of randomized controlled trials and all scored a medium score of quality in the NOS. We used a generalized linear mixed model to pool prevalence rates which is considered to be advantageous over other approaches that are based on transformations (arcsine, and Freeman-Tukey double arcsine), when there are small sample sizes, nevertheless, due to the small number of ACHD patients examined in this systematic review, with various types of disease, firm conclusions cannot be drawn and the results cannot be applied directly in routine clinical practice. Ongoing studies are expected to fill more knowledge gaps regarding the efficacy and safety of NOACs in patients with CHD. A nationwide, prospective, multicenter, observational study is aiming to accumulate 500-patient-years of routine apixaban use in ACHD and atrial arrhythmias until the end of 2022 (NCT03854149). Two interventional prospective randomized controlled trials in Fontan pediatric patients are nearing completion in mid-2020. The first is examining rivaroxaban versus aspirin in 112 patients (NCT02846532); the other is studying 200 patients, to compare 3-arms: apixaban, VKA, and low-molecular-weight heparin (NCT02981472).

## 5. Conclusions

ACHD patients are a heterogeneous population, with often complex pathophysiology requiring specialist care and significant expertise when prescribing anticoagulants. Our systematic review suggests that NOACs are safe and effective in ACHD, even though stronger evidence is needed to confirm this, especially for more complex conditions, such as patients with a Fontan circulation.

## Figures and Tables

**Table 1:** Eligibility Criteria.

Inclusion Criteria	Exclusion Criteria
ACHD patients of any disease severity	Animal Studies
ACHD patients receiving NOAC for: ● Atrial arrhythmias (Intra-atrial reentrant tachycardia, AF, atrial flutter) ● Primary thromboprophylaxis ● Secondary thromboprophylaxis	Pediatric Population
	ACHD patients with severe thrombocytopenia or recurrent hemorrhagic events.

Abbreviations: ACHD: adults with congenital heart disease; AF: atrial fibrillation.

**Table 2:** Quality assessment using the Newcastle-Ottawa scale.

Study	Cohort Representativeness	Selection of Non-Exposed Cohort	Ascertainment of Exposure	The Outcome of Interest Not Present	comparability of Cohorts	Assessment of Outcome	Follow-Up Long Enough	Adequacy of Follow Up of Cohorts	Total Score	Quality of Study
Yang et al. [28]	*	no	*	*	*	*	*	*	7	medium
Pujol et al. [29]	*	no	*	*	no	*	*	*	6	medium
Georgekutty et al. [30]	*	no	*	*	no	*	*	*	6	medium

* high quality choice.

**Table 3:** Specific characteristics of the included studies and their endpoints.

Author	Year	Type of Study	Total Patients	Total Patient-Years of Follow-Up	Primary Endpoint
Yang et al. [28]	2019	Prospective Cohort	530	613	Thromboembolism and major bleeding
Pujol et al. [29]	2019	Retrospective Cohort	215	283.8	Thromboembolism and major, minor bleeding
Georgekutty et al. [30]	2018	Retrospective Cohort	21	26.3	Thromboembolism and major, non-major and minor bleeding

**Table 4:** Descriptive characteristics of included studies.

Author	Male Sex (%)	Age (Years) mean ± SD	Indication for Anticoagulation (%)			Severity of ACHD (%)			Thrombotic Events *n* (%)	Bleeding Events *n* (%)	CHA2ADSVA2Sc ≥ 2 (%)	HAS-BLED ≤ 2 (%)
			AA	PTP	STP	Simple	Moderate	Complex				
Yang et al. [28]	55%	47.0 ± 15.0	90.8	3.2	6.0	14.9	45.1	40.0	6 (1.1)	7 (1.3)	46.4	95.0
Pujol et al. [29]	48%	48.4 ± 15.4	66.8 *	5.6 *	42.9 *	32.1	23.7	44.2	2 (0.7)	9 (3.1)	49.3	87.5
Georgekutty et al. [30]	47%	33.5 ± 8.0	57.0	9.5	33.3	-	-	100	1 (4.76)	0	42.9	100

Abbreviations: AA: atrial arrhythmias; PTP: primary thromboprophylaxis; STP: secondary thromboprophylaxis. * Many patients had more than one indication for anticoagulation treatment (e.g., atrial arrhythmias and stroke/transient ischemic attack).

**Table 5:** Descriptive characteristics of patients with thromboembolic events.

Study	Patients (*n* = 9)	Sex	Age	ACHD Type	NOAC *	Thromboembolic Event	Indication	CHA2DS2-VASc	HAS -BLED	Risk Factors and Comorbidities
Georgekutty [30]	1	M	21	Fontan	Dabigatran 110 mg b.i.d	Deep Vein Thrombosis	Persistent Right-to-Left Shunt	1	0	Protein-losing enteropathy
Yang [28]	2	M	30	Coronary atriovenous fistula	Dabigatran	Deep Vein Thrombosis	Atrial Arrhythmias	3	2	Severe Tricuspid Regurgitation
Yang [28]	3	M	42	Fontan	Apixaban	Pulmonary Embolism	Atrial Arrhythmias	0	3	-
Yang [28]	4	M	25	Fontan	Rivaroxaban	Intracardiac Thrombus	Atrial Arrhythmias	2	1	-
Yang [28]	5	M	44	Tetralogy Fallot	Apixaban	Pulmonary Embolism	Atrial Arrhythmias	1	1	-
Yang [28]	6	M	23	Fontan	Apixaban	Ischemic Stroke	Atrial Arrhythmias	1	0	-
Yang [28]	7	F	25	Transposition of Great Arteries	Apixaban	Intracardiac Thrombus	Atrial Arrhythmias	4	0	Severe Tricuspid Regurgitation
Pujol [29]	8	M	51	VSD (corrected)	Dabigatran 150 mg b.i.d	Stroke	Primary Thromboprophylaxis	3	3	Stroke, TIA, Liver Disease, Arterial Hypertension
Pujol [29]	9	M	50	Aortic Aneurysm	Rivaroxaban 20 mg q.d	Deep Vein Thrombosis	Primary Thromboprophylaxis	1	1	Arterial Hypertension

Abbreviations: VSD: ventricular septal defect; TR: tricuspid valve regurgitation; TIA: Transient ischemic attack; b.i.d: twice a day; q.d: once a day. * NOAC dose indicated where reported.

**Table 6:** Descriptive characteristics of patients with major bleeding events.

Study	Patients (*n* = 16)	Sex	Age	ACHD type	NOAC *	Bleeding Location	Indication	CHA2D2-VASC	HAS-BLED	Risk Factors and Comorbidities
Yang [28]	1	F	56	Fontan	Apixaban	Gastrointestinal	AtrialArrhythmia	2	1	Mitral Regurgitation
Yang [28]	2	F	71	PAPVC	Rivaroxaban	Gastrointestinal	AtrialArrhythmia	3	1	Tricuspid Regurgitation
Yang [28]	3	F	23	CoA	Rivaroxaban	Menorrhagia	AtrialArrhythmia	2	0	Bioprosthetic AVand PV
Yang [28]	4	F	42	Eisenmenger	Rivaroxaban	Menorrhagia	Pulmonary Embolism	3	2	-
Yang [28]	5	F	41	Fontan	Apixaban	Menorrhagia	Atrial Arrhythmia	4	0	Mitral Valve Regurgitation
Yang [28]	6	M	80	ToF	Apixaban	Hematuria	AtrialArrhythmia	4	1	Pulmonary Stenosis
Yang [28]	7	F	67	Fontan	Rivaroxaban	Menorrhagia	AtrialArrhythmia	2	2	-
Pujol [29]	8	M	59	PFO	Apixaban 5 mg b.i.d	Cranial	PTP	3	3	Aspirin Arterial Hypertension
Pujol [29]	9	F	48	TGA-Mustard	Rivaroxaban 20 mg q.d	Cranial	PTP	2	0	Oral Contraception
Pujol [29]	10	M	26	PA + VSD, PH Deletion 22q11	Rivaroxaban 10 mg q.d	Gastrointestinal	PTP	1	3	Renal Insufficiency, Cyanosis, Bleeding under VKA
Pujol [29]	11	M	65	ASD	Apixaban 2.5 mg b.i.d	Intraoccular	PTP	3	2	Arterial Hypertension Smoker
Pujol [29]	12	F	44	PA + VSDPAH	Apixaban 5 mg b.i.d	Intraarticular	PTP	2	0	Renal Insufficiency, Diabetes Mellitus
Pujol [29]	13	F	46	Marfan	Rivaroxaban 20 mg q.d	Intraarticular	PTP	2	0	Smoker
Pujol [29]	14	F	43	ASD	Rivaroxaban 20 mg q.d	Intramuscular	PTP	1	0	-
Pujol [29]	15	F	34	PFO	Edoxaban 60 mg q.d	Vaginal	PTP	3	3	Bleeding under VKA
Pujol [29]	16	F	42	VSD (corrected)	Rivaroxaban 20 mg q.d	Vaginal	PTP	1	0	-

Abbreviations: M: Male; F: Female; PAPVC: partial anomalous pulmonary venous connection; CoA: coarctation of the aorta; ToF: tetralogy of fallot; PFO: persistent foramen ovale; VSD: ventricular Septal defect; ASD: atrial septal defect; PTP: primary thromboprophylaxis; TGA: transposition of great arteries; AV: aortic valve; PV: pulmonary valve; b.i.d: twice a day; q.d; once a day. * NOAC dose indicated where reported.

## Abbreviations

**ACHD**	adult patients with congenital heart disease
**CHD**	congenital heart disease
**VKA**	Vitamin K antagonist
**NOACs**	Non-vitamin K oral anticoagulants
**NOS**	Newcastle-Ottawa scale
**QoL**	Quality of Life

## Appendix A

**MEDLINE search strategy (last search 20/03/2020)**

via PubMed

**#1**

1. Adult congenital heart disease
2. Grown–up congenital heart disease
3. Aortic stenosis
4. Atrial septal defect
5. Atrioventricular septal defect
6. Bicuspid aortic valve
7. Dextrocardia
8. Double inlet left ventricle
9. Double outlet right ventricle
10. Ebstein’s anomaly
11. Hypoplastic left heart syndrome
12. Hypoplastic right heart syndrome
13. Mitral stenosis
14. Persistent truncus arteriosus
15. Pulmonary atresia
16. Pulmonary stenosis
17. Transposition of the great vessels
18. Tricuspid atresia
19. Ventricular septal defect (VSD)
20. New oral anticoagulants
21. NOACs
22. DOACs
23. Xarelto
24. Eliquis
25. Pradaxa
26. Rivaroxaban
27. Apixaban
28. Edoxaban
29. Dabigatran
30. Factor X inhibitors
31. 1 OR 2 OR 3 OR 4 OR 5 OR 6 OR 7 OR 8 OR 9 OR 10 OR 11 OR 12 OR 13 OR 14 OR 15 OR 16 OR 17 OR 18 OR 19
32. 20 OR 21 OR 22 OR 23 OR 24 OR 25 OR 26 OR 27 OR 28 OR 29 OR 30
33. 31 AND 32

**#2**

34. “Factor Xa Inhibitors” [MESH]
35. “Antithrombins” [MESH]
36. “Adult Congenital heart disease” [MESH]
37. 1 OR 2
38. 4 AND 5

**CENTRAL ( 20/03/2020)**

**#1 ** MeSH descriptor: [Heart Defects, Congenital] explode all trees
**#2 ** MeSH descriptor: [Anticoagulants] explode all trees
**#3 ** 1 AND #2
